# Cognitive Fatigue Facilitates Procedural Sequence Learning

**DOI:** 10.3389/fnhum.2016.00086

**Published:** 2016-03-03

**Authors:** Guillermo Borragán, Hichem Slama, Arnaud Destrebecqz, Philippe Peigneux

**Affiliations:** ^1^Neuropsychology and Functional Neuroimaging Research Unit (UR2NF), Centre de Recherches en Cognition et Neurosciences (CRCN), ULB Neurosciences Institute (UNI), Université Libre de Bruxelles (ULB)Brussels, Belgium; ^2^Consciousness Cognition & Computation Group (CO3), Centre de Recherches en Cognition et Neurosciences (CRCN), ULB Neurosciences Institute (UNI), Université Libre de Bruxelles (ULB)Brussels, Belgium

**Keywords:** cognitive fatigue, motor sequence learning, memory competition, serial reaction time (SRT) task, skill learning, procedural learning

## Abstract

Enhanced procedural learning has been evidenced in conditions where cognitive control is diminished, including hypnosis, disruption of prefrontal activity and non-optimal time of the day. Another condition depleting the availability of controlled resources is cognitive fatigue (CF). We tested the hypothesis that CF, eventually leading to diminished cognitive control, facilitates procedural sequence learning. In a two-day experiment, 23 young healthy adults were administered a serial reaction time task (SRTT) following the induction of high or low levels of CF, in a counterbalanced order. CF was induced using the Time load Dual-back (TloadDback) paradigm, a dual working memory task that allows tailoring cognitive load levels to the individual’s optimal performance capacity. In line with our hypothesis, reaction times (RT) in the SRTT were faster in the high- than in the low-level fatigue condition, and performance improvement was higher for the sequential than the motor components. Altogether, our results suggest a paradoxical, facilitating impact of CF on procedural motor sequence learning. We propose that facilitated learning in the high-level fatigue condition stems from a reduction in the cognitive resources devoted to cognitive control processes that normally oppose automatic procedural acquisition mechanisms.

## Introduction

Animal studies (e.g., White and McDonald, 2002) and clinical evidence in humans (e.g., ; Heindel et al., 1989; Tranel et al., 1994) show that memory is not a unitary phenomenon. Rather, it is best understood as the result of a combination of different systems or brain processes that either operate in parallel or enter in competition. On one hand, memory systems can cooperate in a compensatory way (e.g., during route recognition in Huntington disease, hippocampus activity can compensate for the gradual dysfunction of the caudate nuclei; Voermans et al., 2004). On the other hand, brain systems can interact in a competitive relationship (Hartley and Burgess, 2005) to access and integrate information in such a way that disabling one system gives free rein to another to mediate the learning process (for a review see Krupa, 2009). For instance, there is a competitive relationship between the striatal and medial temporal lobe (MTL) regions, in such a way that implicit memory performance is better when striatal activity is high and MTL activity is low; and conversely explicit memory performance is better when MTL activity increases and striatal activity diminishes (Poldrack et al., 2001). Likewise, there is a negative coupling between the activity of the anterior cingulate/medial prefrontal cortex and the striatum during explicit but not implicit memory retrieval (Destrebecqz et al., 2005). Since implicit memory was exclusively associated with striatal activity in this latter study, it is suggested that the influence of implicit processes can be successfully controlled by conscious knowledge during explicit memory retrieval.

The competition between the prefrontal cortex and basal ganglia systems has also been invoked for the control of behavior (Daw et al., 2005). In this framework, experimental manipulations that reduce the efficiency of executive control and attentional systems, e.g., the disruption of dorsolateral prefrontal cortex activity by transcranial magnetic stimulation (TMS; Galea et al., 2010; Smittenaar et al., 2013), hypnosis (Nemeth et al., 2013) or increased working memory demands (Filoteo et al., 2010) have been shown to enhance consolidation and the acquisition of procedural skills. Altogether, these studies suggest that learning in a particular memory system is facilitated under circumstances in which the expression of other competing memory systems is hampered.

Another condition depleting the availability of controlled resources is mental or cognitive fatigue (CF), defined as the decrease in cognitive resources developing over time on sustained cognitive demands independently of sleepiness (Trejo et al., 2005). CF is associated with impaired cognitive control (Lorist et al., 2005), high-level information processing (Tanaka et al., 2012) and sustained attention (Langner et al., 2010). Exposure to High Cognitive Load (HCL) levels, conditions where the time to process ongoing cognitive demands is restricted, also leads to increased CF (Borragán et al., submitted). Magnetoencephalographic data suggest that impaired activity in the anterior cingulate and dorsolateral prefrontal cortical regions triggers the subjective feeling of CF and the decision to rest (Ishii et al., 2014). Accordingly, arterial spin labeling perfusion fMRI has evidenced deactivation in the fronto-parietal network during rest after sustained mental workload (Lim et al., 2010). In this framework, CF might directly diminish available cognitive reserves and facilitate the disengagement of resources consuming controlled top-down memory systems. Hence, reduced goal-directed attention with CF would eventually lead to stimulus-driven performance (Boksem et al., 2005).

In the present study, we tested the hypothesis that CF would facilitate performance in automatic, procedural forms of learning that do not require, or are potentially hampered by, controlled cognitive resources. To do so, we investigated whether triggering high levels of CF may enhance acquisition performance in a motor procedural serial reaction time (SRT) task. At the neuroanatomical level, we assumed that mostly basal ganglia activity would subtend learning in the high CF condition, considering that high CF deplete the fronto-parietal resources underlying attentional and executive functions (Lorist et al., 2005; Lim et al., 2010; Ishii et al., 2014). More specifically, we reasoned that triggering high levels of CF before learning would hamper the prefrontal executive resources competing with subcortical activity and support the controlled declarative memory component of the task. Indeed, striatal activity supports habit formation (Yin and Knowlton, 2006) and implicit sequence learning (Destrebecqz et al., 2005), and increasing the working memory load biases neural competition in favor of habit memory mechanisms (Foerde et al., 2006). As a result, implicit motor procedural learning that mainly relies on striatal activity should develop better.

## Materials and Methods

### Participants

Twenty-three French-speaking participants (17 women, 4 left-handed; mean age ± SD 23.04 ± 4.14 years) without any history of psychiatric or neurological disease gave their written, informed consent to participate in the present study conducted in accordance with the Declaration of Helsinki and approved by the Ethics Committee of the Faculty of Psychological Sciences (Université Libre de Bruxelles, ULB). All participants had normal to acceptable sleep quality in the past month (Pittsburgh Sleep Quality Index score <7; Buysse et al., 1989). Participants also exhibited moderate to neutral chronotype (31 > morningness–eveningness questionnaire score < 70; Horne and Ostberg, 1976).

### Material and Tasks

#### Cognitive Fatigue Induction: Time load Dual-back (TloadDback Task)

The Time load Dual-back (TloadDback; Borragán et al., submitted) is a task in which different levels of cognitive load can be induced and individually adjusted by modifying the time available to process and manipulate the ongoing task demands. Basically, the TloadDback task is a dual task featuring a classical N-back working-memory updating task (Kirchner, 1958) and a parity number decision task. Digits and letters are displayed in alternation on the screen, and participants are instructed to press the space bar with their left hand every time the displayed letter is the same as the penultimate letter, or to indicate whether the displayed digit is odd or even by pressing “1” or “2” on the numeric keypad. Combining two tasks featuring different requirements for information processing ensures a large recruitment of working memory resources, an involvement that can be adjusted with the pace at which the information is processed. During a pre-test session, the maximal load level (i.e., the fastest stimulus time duration (STD) allowing accuracy performance >85%) is determined separately for each participant. This maximal load level corresponds to the HCL condition. In the Low Cognitive Load (LCL) condition, stimulus presentation rate is made 1/3 slower [i.e., STD (LCL) = STD (HCL) + 1/2 STD (HCL)]. Hence, both LCL and HCL conditions have the same level of complexity, but the available processing time is proportionally different and tailored to each participant’s processing capacity. The duration of the task is 16 min. The evolution of CF is assessed: (a) at the subjective level using a Visual Analogue Scale for fatigue (VASf; Lee et al., 1991) before and after the TloadDback task; and (b) objectively by computing the evolution of performance within the TloadDback task (Lorist et al., 2000; van der Linden et al., 2003; Campagne et al., 2004; Faber et al., 2012). Performance levels are computed over four successive time periods (t1, t2, t3, t4) including each ± 20% of the total number trials.

#### Procedural Learning: Serial Reaction Time Task

We used a tactile variant of the classical Serial Reaction Time Task (SRTT; Nissen and Bullemer, 1987). In this version (Borragán et al., 2015), stimuli (i.e., the drawing of a car) were presented using E-Prime Software (Psychology Software Tools) at one out of the four corners on a computer screen (16 inches; refresh rate of 60 Hz) adapted for tactile responses (Magic Touch Add-On Touch Screen, KeyTec-Inc.). Participants were instructed to press the location of the stimulus with their right hand as quickly and as accurately as possible. The stimulus remained on the screen until subject’s response, with the next stimulus being displayed immediately after the response (response stimulus interval [RSI] = 0 ms). The learning session consisted of eight blocks (B1 to B8; 96 stimuli/block) for an approximate duration of 6–7 min. Unbeknownst to participants, a fixed 12-element sequence of positions (**A**: 1, 4, 2, 1, 3, 2, 4, 1, 3, 4, 2, 3 or **B**: 3, 2, 4, 1, 3, 4, 1, 2, 4, 3, 1, 4) was repeated during six blocks (B2 to B6 and B8). In blocks B1 and B7, the succession of positions was pseudo-random. Trills (e.g., 1, 2, 1), runs (e.g., 1, 2, 3, 4) and repetitions (e.g., 1, 1) are excluded both in regular sequences A and B and in pseudo-random sequences (Goedert and Willingham, 2002). Sequence A (respectively, B) was used for SRT learning on day 1, and sequence B (respectively, A) for SRT learning on day 2, in a counterbalanced order.

#### Sequence Generation Task

At the end of the experimental session on day 2, participants were informed about the presence of a repeated sequence of stimuli in the majority of the SRTT blocks, and that their knowledge about the regularities of the sequence practiced on day 2 will be assessed in a generation task (Destrebecqz and Cleeremans, 2001). The generation task is an adaptation to sequence learning of the process dissociation procedure (PDP; Jacoby, 1991). It aims at providing a measure of how implicit and explicit memory components contribute to performance in a single task. In the Inclusion condition, participants have to reproduce the learned sequence of stimuli by pointing to the successive positions on the tactile screen for 96 trials (i.e., 1 block). If they claim having no explicit memory of the sequence, they are encouraged to follow their best feeling. Hence, generation performance can be due both to explicit and implicit knowledge in this Inclusion condition. Contrarily, in the Exclusion condition, participants are asked to generate a sequence of positions that is different from the learned sequence, also for 96 trials. In this case, continued generation of learned elements in spite of the exclusion instructions indicate a lack of conscious knowledge, as participants are unable to prevent producing familiar elements. Inclusion and Exclusion condition order was counterbalanced between subjects.

Generation performance in the Exclusion and Inclusion conditions is computed as the percentage of generated triplets (or chunks) belonging to the learned sequence (i.e., maximal 100% score is obtained with 94 correctly generated triplets out of 94, as the total number of stimuli is 96). Chance level is 33%. In addition, an index of explicit knowledge is calculated by computing the difference between inclusion and exclusion scores (I-E). A higher index signifies a higher level of explicit knowledge and conscious control over the learned sequence (for details, see Destrebecqz et al., 2005).

#### Procedure

**Figure 1:**
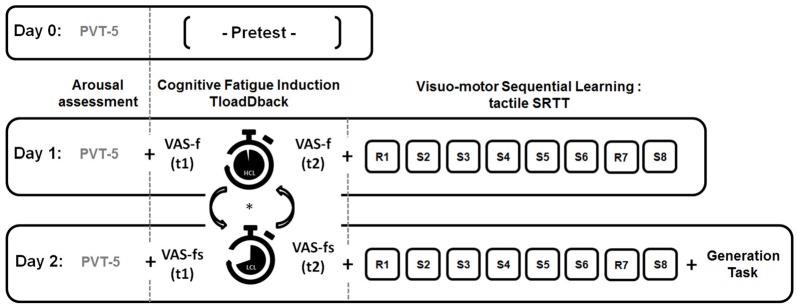
**Experimental design.** On Day 0, participants are administered a pre-test to determine their maximal cognitive load capacity on the TloadDback task (i.e., fastest pace allowing accuracy performance >85%) . On Day 1 and Day 2, they perform the TloadDback task either in a High Cognitive Load (HCL) or in a Low Cognitive Load (LCL) condition, in counterbalanced order. Immediately before and after completion of the TloadDback task, participants complete the Visual Analogue Scale of fatigue (VAS-f). They are then administered the Serial Reaction Time Task (SRTT) using either repeated sequence A or B, in counterbalanced order. Additionally, at the end of the Day 2 session, they are asked to accomplish a generation task to test their knowledge about the sequential patterns in the last learned sequence. Vigilance levels prior to the beginning of the experiment are measured every day using a psychomotor vigilance task (PVT-5). Each session lasted approximately 30 min.

Our experimental design is illustrated Figure 1. To ensure similar levels of vigilance over the 3 days of the experiment, a 5-min version of the psychomotor vigilance task (PVT; Dinges and Powell, 1985) was administered at the beginning of each session. On day 0, a pretest session determined the maximal cognitive load capacity for each participant through the TloadDback task. On day 1, the TloadDback task was administered either in the HCL or the LCL condition (the order was counterbalanced between participants). Subjective evolution of CF was calculated by subtracting the VASf scores before the TloadDback from the VASf scores after. Immediately after the TloadDback task, participants were administered the SRTT learning session using either sequence A or B (counterbalanced). The same procedure was repeated on day 2 using the other SRTT sequence (B or A). Finally, the generation task was administered at the end of day 2.

## Results

### Sleep Quality and Baseline Vigilance Levels Within the Experiment

Sleep duration and sleep quality for the nights preceding the testing sessions did not differ significantly from each other (*p*s > 0.7). Mean ± standard deviation for sleep duration and sleep quality were Night 0 = 7.63 ± 1.45 h and 4.5 ± 0.9; Night 1 = 7.62 ± 0.94 h and 4.32 ± 0.8; Night 2 = 7.93 ± 1.17 h and 4.45 ± 0.8 (as derived from the St-Mary Hospital Questionnaire, Ellis et al., 1981).

A repeated-measures ANOVA conducted on Reciprocal Reaction Times on the PVT (i.e., mean 1/RT; Basner and Dinges, 2011) with Day (D0, D1 and D2) as the within-subject factor was not significant (*F*_(1,22)_ = 0.14, *p* > 0.86; *M ± SD* Day 0 = 0.3 ± 0.02, Day 1 = 0.3 ± 0.03 and Day 2 = 0.31 ± 0.03), which did not support the assumption of differences in vigilance levels between the experimental sessions.

### Induction of Cognitive Fatigue

**Figure 2:**
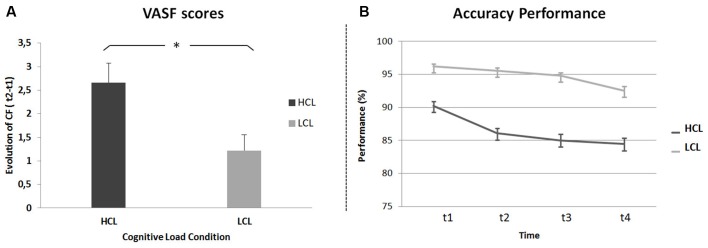
**Cognitive Fatigue (CF). (A)** Task-related CF (difference between VAS-f scores before and after the TloadDback task) in high (HCL) and low (LCL) cognitive load conditions. **(B)** Evolution of performance (accuracy scores) across four quartiles (± 3 min each) during the TloadDback in the HCL and the LCL conditions. Error bars represent standard errors.

For subjective measures, a repeated-measures ANOVA was run on CF scores (i.e., the difference between VAS-fatigue (VAS-f) scores before (t1) and after (t4) the TloadDback task) with Cognitive Load (HCL and LCL) as the within-subject factor and condition administration Order (HCL then LCL vs. LCL then HCL) as the between-subjects factor. Results disclosed a main effect of Cognitive Load (*F*_(1,21)_ = 8, *p* < 0.05, *MSE* = 2.90; *η*^2^ = 0.27), with higher CF in the HCL (VAS-f score 2.66 ± 2) than the LCL (1.22 ± 1.59) condition (Figure 2A). The analysis did not show any other significant effect or interaction (all *p* values > 0.45).

To investigate the evolution of CF during the TloadDback task, a repeated-measures ANOVA was computed on weighted accuracy scores with Cognitive Load condition (HCL vs. LCL) and Time on Task (t1 vs. t2 vs. t3 vs. t4) as within-subject factors and administration Order (HCL-LCL vs. LCL-HCL) as the between-subjects factor. The analysis disclosed a main effect of Cognitive Load (*F*_(1,20)_ = 24.3, *p* < 0.001; *MSE* = 1; *η^2^* = 0.55) with higher performance levels during the LCL (94.8 ± 1.6%) than the HCL (86.4 ± 2.62%) condition, although performance was above the required accuracy level (i.e., 85%) in both conditions. The Time on Task effect was also significant (*F*_(3, 60)_ = 9.14, *p* < 0.001; *MSE* = 0.16; *η^2^* = 0.31). *Post hoc* tests revealed higher performance at the beginning than at the end of practice (93.2 > 90.8 > 89.9 > 88.5%; t1 > (t3 = t4) and t2 > t4; *p*s < 0.01; Figure 2B). Finally, the Cognitive Load by Time on Task interaction was significant (*F*_(3,60)_ = 3.6, *p* < 0.05; *MSE* = 0.10; *η^2^* = 0.12), indicating a different evolution of performance in the HCL and LCL conditions. As illustrated in Figure 2B, performance decreased in both the HCL and LCL conditions from the beginning to the end of the task (t1 > t4; Tukey’s *post hoc* *ps* < 0.05), but decreased faster in the HCL (t1 > (t2 > (t3 = t4))) than in the LCL condition ((t1 = t2 = t3) > t4). These results suggest that, as expected, cognitive demands and resulting CF were higher in the HCL condition. All other effects were non-significant (all *p* values > 0.4).

### Serial Reaction Time Task (SRTT)

**Figure 3:**
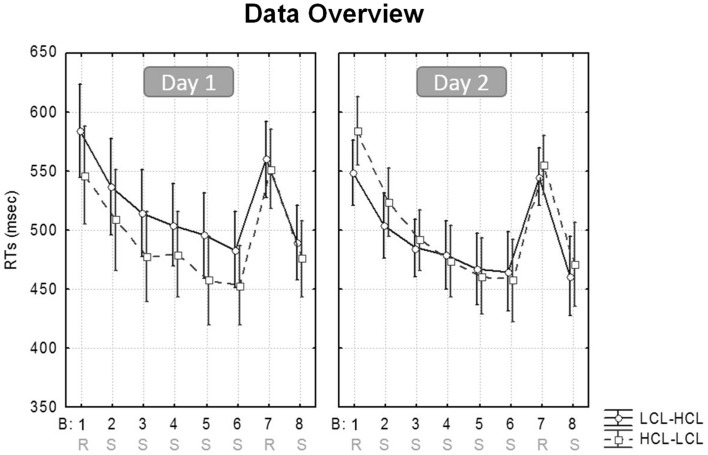
**SRTT performance.** Mean RT/block in the high (HCL) and low (LCL) cognitive load conditions (HCL vs. LCL), for participants learning on Day 1 in the LCL then on Day 2 in the HCL condition (LCL–HCL), and participants learning first in the HCL then in the LCL condition (HCL–LCL).

Reaction times (RTs) for only correct responses were averaged per block (Borragán et al., 2015). RTs >3 standard deviations from the mean were excluded, and responses given outside of the stimulus target (the 5 × 6 cm^2^ at each corner of the screen) were considered as errors. Analyses conducted on accuracy scores only disclosed slightly more errors in the Random than in the Sequential blocks (3.13 ± 1.4% vs. 3.74 ± 2.38%; (*F*_(1,19)_ = 22.7, *MSE* = 0.76; *p* < 0.001; *η*^2^ = 0.54), with no interaction involving any other factors (all *p*s > 0.16). Therefore, and given the low proportion of errors, subsequent analyses were only computed for RTs. Figure 3 illustrates the evolution of speed performance in the two experimental conditions (HCL and LCL) during the two successive days.

As a reminder, our analyses aimed at investigating whether sequence learning is enhanced after a high level of CF is induced (HCL condition), as compared to after a low level of CF (LCL condition). A repeated-measures ANOVA was conducted on response speed (mean RT/block) with Cognitive Load (HCL vs. LCL), Block Type (Sequential vs. Random) and Task Practice (i.e., Beginning [Blocks 1–2] vs. End [Blocks 7-average (6/8)] of the learning session) as within-subject factors, and Sequence (A vs. B) and Condition Order (HCL-LCL vs. LCL-HCL) as between-subjects factors. Note that Sequential blocks 6 and 8 were averaged to obtain a more accurate measure of performance at the end of the learning session in comparison with the intermediate Random block 7 (Borragán et al., 2015).

**Figure 4:**
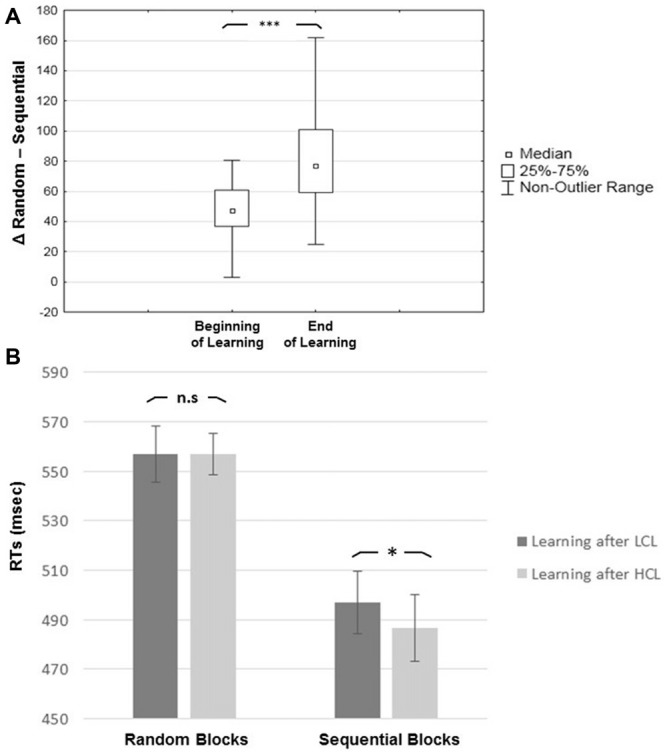
**Learning effects. (A)** Learning effect (RTs for random minus sequential blocks). **(B)** Type of Block by Condition interaction, showing that the positive effect of CF is present for sequential but not random blocks. Error bar represent one standard deviation from the mean****; Asterisks indicate *p*-value significance after Tukey *post hoc* correction: *p < 0.05; **p < 0.01 and ***p < 0.001. n.s: non-significant.

In looking at learning effects, results revealed a main effect of Block Type (*F*_(1,19)_ = 149.61, *MSE* = 1234; *p* < 0.001; *η^2^* = 0.89), with slower RTs for Random (557 ± 44.83 ms) than for Sequential (492 ± 46 ms) blocks, indicating an advantage of the repeated sequence (i.e., a learning effect). There was also a main effect of Task Practice (*F*_(1,19)_ = 42.06, *MSE* = 1063; *p* < 0.001; *η^2^* = 0.69), with faster RTs at the end (508 ± 41 ms) than at the beginning (540 ± 48 ms) of the SRTT session. The interaction between Block Type and Task Practice was significant (*F*_(1,19)_ = 33.06, *MSE* = 380; *p* < 0.001; *η^2^* = 0.63): RT differences between Sequential and Random blocks were higher at the end (83 ± 33 ms) than at the beginning (47 ± 21 ms) of the SRTT session (*p* < 0.001), indicating a progressive learning of the sequential regularities (see Figure 4A).

Regarding the effect of CF, the main effect of Cognitive Load was non-significant (*p* > 0.48) but there was a significant interaction between Cognitive Load and Block Type (*F*_(1,19)_ = 4.46, *MSE* = 252; *p* < 0.05; *η*^2^ = 0.19). *Post hoc* tests disclosed significantly faster RTs in the HCL than the LCL condition for Sequential (487 ± 46 ms vs. 497 ± 53 ms; *p* < 0.05) but not for Random blocks (556 ± 41 ms vs. 557 ± 54 ms; *p* > 0.76; Figure 4B), suggesting that CF mostly had a positive impact on performance for the sequential component of procedural learning in the SRTT. Also, the interaction between Cognitive Load and Condition Order factors was significant (*F*_(1,19)_ = 6.59, *MSE* = 2324; *p* < 0.02; *η*^2^ = 0.26). *Post hoc* tests revealed faster RTs that were marginally significant (irrespective of Sequential or Random blocks) with regard to only high CF for participants who received the LCL condition first (LCL vs. HCL = 536 ± 71 ms vs. 512 ± 57 ms; *p* = 0.06). This was not the case for participants who completed the HCL condition first (HCL vs. LCL = 532 ± 60 ms vs. 518 ± 76 ms, *p* > 0.54).

### Generation Task

Exclusion generation scores (% of chunks belonging to the sequence learned on day 2) were above chance level in the HCL [single-sample *t*-test against 33% value, *t*_(12)_ = 3.13, *p* < 0.02] but not in the LCL (*p* > 0.1) condition. This suggests that participants in the LCL condition had more control and explicit knowledge about the learned sequence than participants in the HCL condition. However, a one-way ANOVA computed for generation scores with Instruction (Inclusion vs. Exclusion) as the within-subject factor and Cognitive Load (HCL vs. LCL) as between-subjects factor failed to reveal main or interaction effects (all *p*s > 0.3). Additionally, the index of explicit knowledge (inclusion minus exclusion scores) did not significantly differ from zero in either the HCL (*p* > 0.35) or LCL (*p* > 0.5) conditions, suggesting a lack of conscious knowledge about the regularities embedded in the sequential material.

## Discussion

The present study aimed at exploring a paradoxical, facilitating effect of CF due to prior exposure to HCL on procedural sequence learning in a SRTT. CF was successfully elicited in our experiment. Indeed, in comparing the HCL with the LCL condition, subjective fatigue (VASf) scores showed more of an increase in the HCL condition, with accuracy performance during the TloadDback task being lower and decreasing more rapidly. As expected, there was sequence learning in both CF conditions, with faster RTs for repeated than for random sequences of stimuli. Importantly, the improved performance for repeated and not random sequences from increased CF levels indicates that the facilitating effect of CF is restricted to the sequential component of motor sequence learning. Finally, performance in the generation task indicates that learning in the SRTT remained essentially implicit in both LCL and HCL conditions, although analysis of exclusion scores suggests less top-down control about sequential knowledge at high CF levels. These results corroborate the proposal that facilitative learning effects on one memory system may stem from the disengagement of another competing memory system (Foerde et al., 2006; Brown and Robertson, 2007a,b).

Prior studies have already reported enhanced procedural learning in conditions where cognitive control is reduced (Foerde et al., 2006; Filoteo et al., 2010; Galea et al., 2010; Nemeth et al., 2013; Delpouve et al., 2014). To the best of our knowledge, the present study is the first to report a facilitation of procedural learning after increased CF due to prior exposure to HCL levels. In this framework, CF might be a factor that directly diminishes available cognitive reserves, and eventually facilitates the disengagement of the controlled top-down memory systems that are demanding in terms of cognitive resources. This proposal is in agreement with the view that mental or CF as a reduction in goal-directed attention eventually leading to performing in a stimulus-driven fashion (Boksem et al., 2005). In the present study, we hypothesize that it is essentially activity in the basal ganglia that supported the learning process in the high CF condition, assuming that high CF levels had actually depleted the fronto-parietal resources that underlie attentional and executive functions (Lorist et al., 2005; Lim et al., 2010; Ishii et al., 2014). Indeed, striatal activity, which is associated with habit formation (Yin and Knowlton, 2006) and automatic detection of complex regularities (Peigneux et al., 2000), supports the implicit processing of sequential patterns (Destrebecqz et al., 2005). Furthermore, increasing the working memory load actually biases the competition in favor of habit memory mechanisms (Foerde et al., 2006). Accordingly, we used the TloadDback task to saturate working memory resources for a period of time in order to induce CF (Borragán et al., submitted). Notably however, we demonstrated the aftereffects of sustained cognitive load in terms of persistent CF here, which reflects a temporary inability to regain the sufficient cognitive resources to drive top-down controlled processes during the learning episode. Notwithstanding, we recognize that a limitation of the present study is the lack of brain activity recordings to support the functional hypotheses. Future neuroimaging studies should address this issue of an imbalance between the neural substrates of competing memory systems in different CF conditions. Additionally, our participants were healthy young adults, and it is unclear how cognitive performance is modulated by fatigue as a function of age. Although the topic is still barely explored, and was beyond the scope of the present study, we argue that individual adjustment to each participant’s maximal cognitive load in the TloadDback task normalizes for a possible effect of age. Indeed, a different, adjusted cognitive load would be defined for older or younger participants as a function of their capacity, thus equating cognitive demands. Notwithstanding, future studies should test whether CF and its effects evolve with age even in controlled cognitive load conditions.

Our results show that CF is specifically beneficial for the acquisition of the sequential components in the SRTT, but not the motor learning components (i.e., performance in random blocks). Additionally, the analysis of exclusion scores in the generation task suggests that participants performed slightly better in repeating learned sequential patterns in the LCL than in the HCL condition. This suggests less control over the learned sequence in the HCL condition. Together with the finding of faster RTs for sequential blocks in the HCL condition, these results are in agreement with the proposal that learning was more automatic in this resource-depleting condition. Also in line with this proposal, other studies have shown that testing participants at their non-optimal time of the day (i.e., when they feel the least ability to perform cognitively demanding tasks) is actually associated with an increased performance in implicit learning and procedural memory (May et al., 2005; Delpouve et al., 2014), whereas performance deteriorates in an explicit memory task (May et al., 2005). We show that, independently of time-of-day, which was a random factor in this study, previous cognitive demands and the ensuing CF influence the relative involvement of controlled and automatic memory systems on performance in a SRT task. Notably, our results cannot be explained by sleep disturbances known to trigger CF (Akerstedt et al., 2004), and vigilance levels were similar during pre-testing and both HCL and LCL conditions in this within-subject design.

Cooperative and competitive interactions among different memory systems is a currently developing topic of interest in the cognitive neurosciences. Whereas some memory systems exhibit dependency relationships, others might act more independently under certain circumstances (Klein et al., 2002; Voermans et al., 2004; Hartley and Burgess, 2005), which might represent an adaptive and evolutionary competitive mechanism (Klein et al., 2002) that remains to be fully understood. Presently, it has come to be recognized that competitive relationships in memory systems are dynamic in nature and are modulated by various factors, such as the presence or absence of sleep during the consolidation period (Orban et al., 2006; Brown and Robertson, 2007b; Albouy et al., 2008, 2013; Rauchs et al., 2008) and available resource levels (Foerde et al., 2006; Filoteo et al., 2010), that can themselves be associated with CF.

To conclude, our results challenge the idea that CF results only in negative consequences on cognition. Aside from representing a useful signal that cognitive resources are saturated and that there is a need for rest and/or change of activity, CF may also modify the balance between memory systems in such a way that it facilitates the automatic acquisition of novel skills. Finally, our results stress the need to consider CF as a moderating factor in learning and memory performance and that the impact of CF on the different cognitive components involved in a given task should be assessed separately.

## Author Contributions

GB: tested conceptualization of the hypothesis. He conducted the experimental testing and the statistical analysis. Finally, he wrote the article. HS: tested conceptualization of the hypothesis. AD: methodological advice. PP: tested conceptualization of the hypothesis and statistical analysis.

## Conflict of Interest Statement

The authors declare that the research was conducted in the absence of any commercial or financial relationships that could be construed as a potential conflict of interest.
